# Loss of *C*. *elegans* GON-1, an ADAMTS9 Homolog, Decreases Secretion Resulting in Altered Lifespan and Dauer Formation

**DOI:** 10.1371/journal.pone.0133966

**Published:** 2015-07-28

**Authors:** Sawako Yoshina, Shohei Mitani

**Affiliations:** 1 Department of Physiology, Tokyo Women’s Medical University School of Medicine, Tokyo, 162–8666, Japan; 2 Tokyo Women’s Medical University Institute for Integrated Medical Sciences (TIIMS), Tokyo Women’s Medical University, Tokyo, 162–8666, Japan; Brown University/Harvard, UNITED STATES

## Abstract

ADAMTS9 is a metalloprotease that cleaves components of the extracellular matrix and is also implicated in transport from the endoplasmic reticulum (ER) to the Golgi. It has been reported that an ADAMTS9 gene variant is associated with type 2 diabetes. The underlying pathology of type 2 diabetes is insulin resistance and beta-cell dysfunction. However, the molecular mechanisms underlying ADAMTS9 function in beta cells and peripheral tissues are unknown. We show that loss of *C*. *elegans* GON-1, an ADAMTS9 homolog, alters lifespan and dauer formation. GON-1 loss impairs secretion of proteins such as insulin orthologs and TGF-beta, and additionally impacts insulin/IGF-1 signaling in peripheral tissues. The function of the GON domain, but not the protease domain, is essential for normal lifespan and dauer formation in these scenarios. We conclude that the GON domain is critical for ADAMTS9/GON-1 function across species, which should help the understanding of type 2 diabetes in humans.

## Introduction

Type 2 diabetes is a multifactorial disease characterized by impaired insulin secretion and insulin resistance. The risks for and progression of type 2 diabetes are determined by a combination of genetic and environmental factors. Recently, more than 60 common type 2 diabetes risk variants were identified through genome-wide association studies (GWAS) [1]. It was reported that an ADAMTS9 gene variant found in the 5'-upstream region (rs4607103) is associated with type 2 diabetes [2, 3]. Numerous studies have raised the possibility that this ADAMSTS9 gene variant is associated with insulin resistance and beta-cell function. However, the molecular mechanisms underlying how ADAMTS9 affects beta cells and peripheral tissues are unknown. In mice, an ADAMTS9 null allele is lethal in early embryonic stages [4]. The phenotype of the ADAMTS9 gene variant (rs4607103) is milder, perhaps because this allele is a weak reduction-of-function ADAMTS9 allele.

Insulin signaling is highly conserved between *C*. *elegans* and humans. In *C*. *elegans*, the insulin/insulin-like growth factor (IGF) receptor DAF-2 signals through the PI3-kinase [5] signaling cascade, which activates downstream serine/threonine kinases [6]. These kinases negatively regulate FOXO/DAF-16. The insulin signaling pathway has been shown to regulate developmental processes, such as dauer formation, lifespan and behavior [5, 7–9]. In the presence of food, worms progress through larval stages to the adult stage, whereas in the absence of food, worms halt development at the dauer larval stage. Because of fundamental similarities in the insulin signaling pathway between worms and humans, genetic and molecular pathways that control metabolism in the worm could be highly informative for analysis of human metabolic diseases such as type 2 diabetes.

ADAMTS9 is a metalloprotease that cleaves components of the extracellular matrix (ECM) [10, 11], and it is also implicated in transport from the endoplasmic reticulum (ER) to the Golgi [12]. Downregulation of GON-1, the *C*. *elegans* homolog of ADAMTS9, results in the inhibition of protein transport from the ER to the Golgi [12]. Because a variant of ADAMTS9 has been established as a risk factor for type 2 diabetes [1], we investigated how ADAMTS9/GON-1 is involved in the insulin/IGF-like signaling pathway. We show that both insulin secretion and the insulin/IGF-like signaling pathway are affected by GON-1 depletion and recovered by GON domain expression.

## Results

### GON-1 is important for insulin secretion

**Fig 1:**
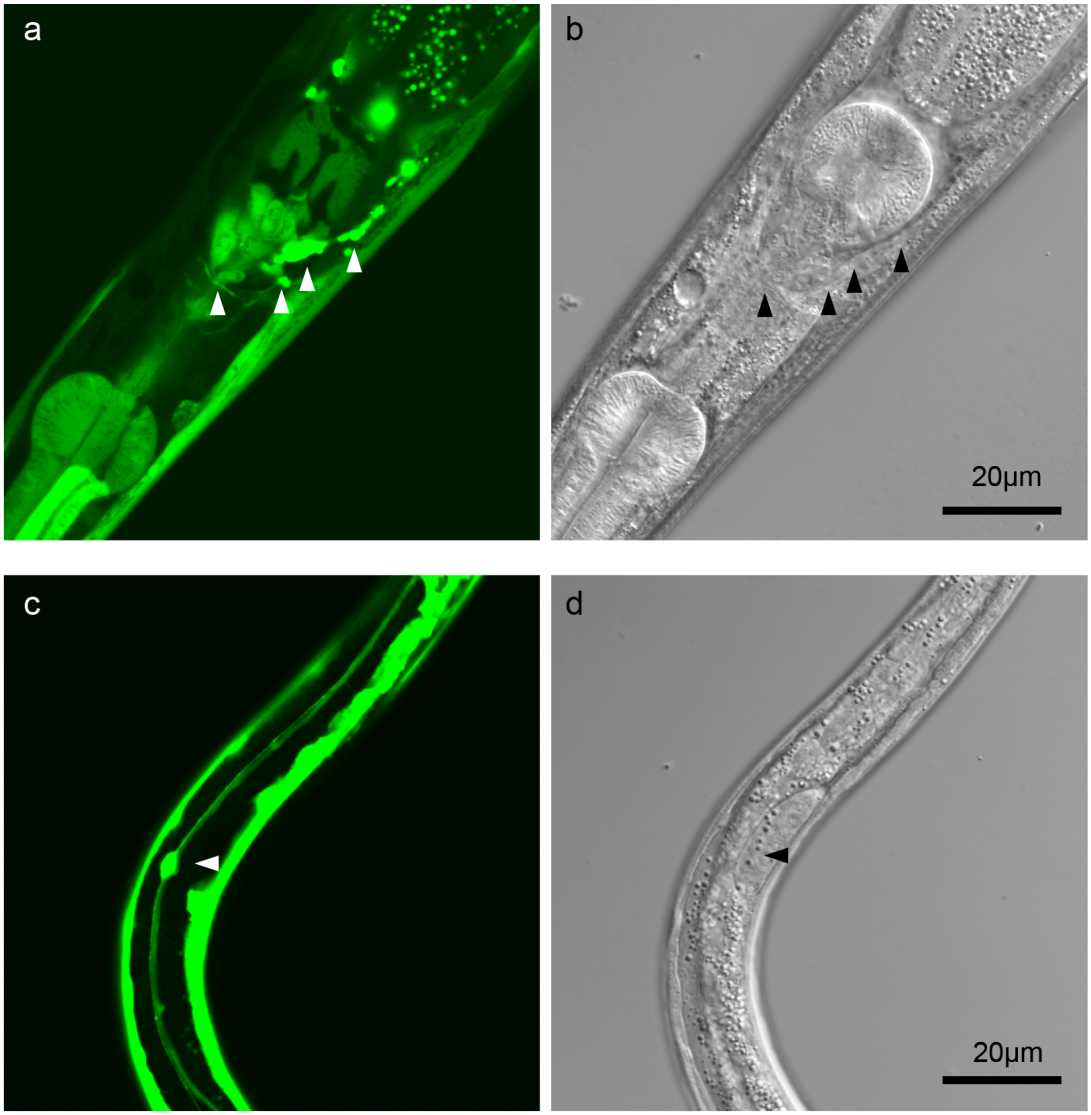
*gon-1* is expressed in a subset of neurons. (a, c), GFP fluorescence; (b, d), DIC image. (a) GFP is observed in some head neurons (arrowheads), intestine, and excretory cells in the larva. (c) GFP is observed in a CAN neuron (arrowhead), an excretory canal cell, and body wall muscle cells.

In *C*. *elegans*, GON-1 has been reported to be expressed in distal tip cells (DTCs) and body wall muscle cells, and it functions in ECM remodeling and the protein secretory pathway. Depletion of GON-1 results in defects in DTC migration [13] and protein secretion [12]. To determine whether GON-1 is involved in other phenomena, we evaluated whether *gon-1* is expressed in other cells in addition to DTCs and body wall muscle cells. *gon-1* is expressed in certain additional cell types at the larval and adult stages, including neuronal, intestinal, and excretory cells. *gon-1* is also expressed in certain cells at the embryonic stage (Fig 1 and S1 Fig).

**Fig 2:**
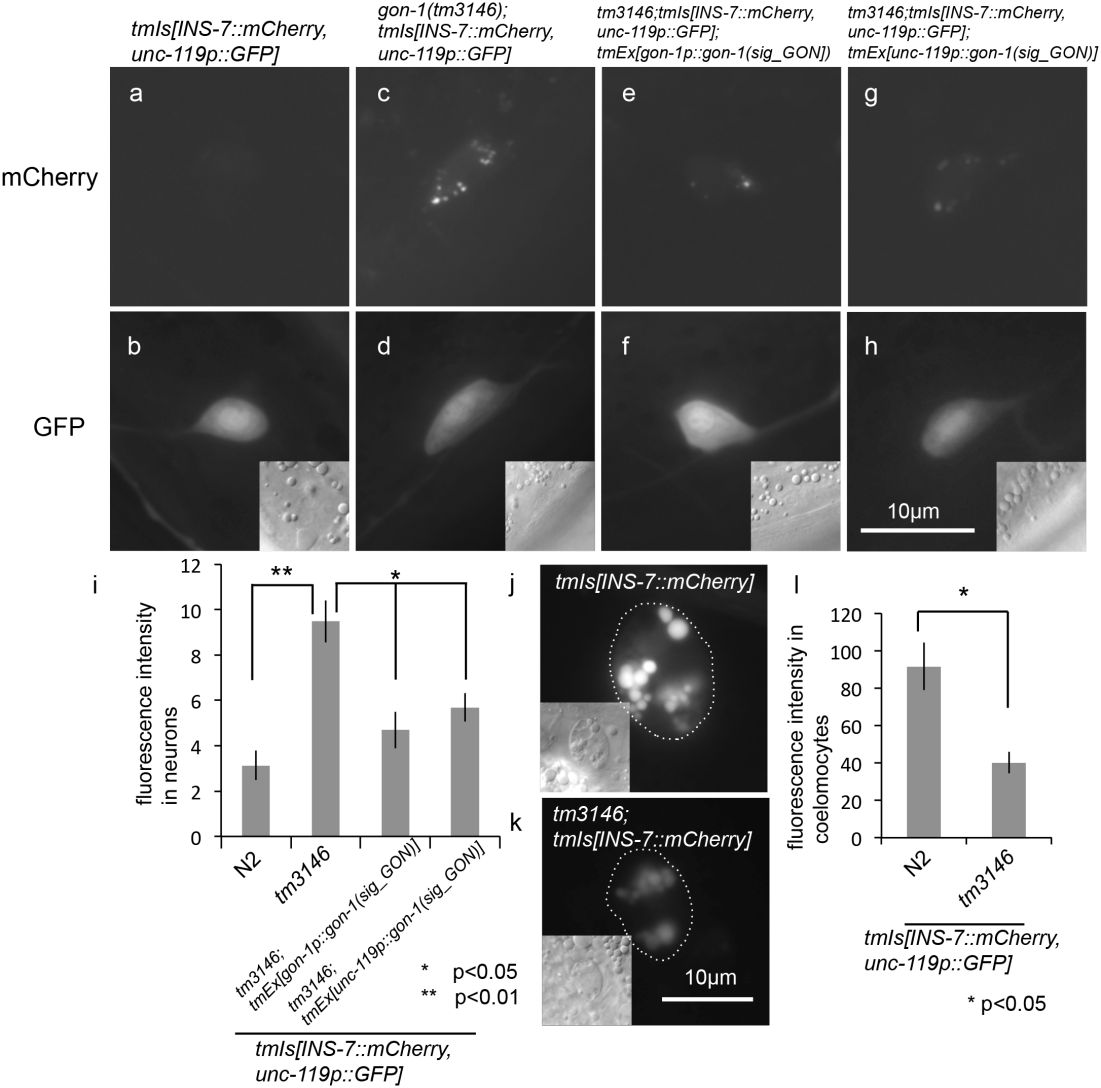
Depletion of *gon-1* causes INS-7 secretion defects. Representative fluorescent images of INS-7::mCherry (a, c, e, g) (500 msec. exposure time), *unc-119p*::*GFP* (b, d, f, h) and DIC images (inset) in *ins-7* expressing neurons. (a, b) Wild-type. (c, d) *gon-1(tm3146)*. The accumulation of INS-7::mCherry was observed in some neurons in the *gon-1(tm3146)* mutant background. (e, f) *gon-1(tm3146); tmEx[gon-1p*::*gon-1(sig_GON)]* animals. (g, h) *gon-1(tm3146); tmEx[unc-119p*::*gon-1(sig_GON)]* animals. The accumulation of INS-7::mCherry was scarcely observed in *gon-1(tm3146); tmEx[gon-1p*::*gon-1(sig_GON)]* and *gon-1(tm3146); tmEx[unc-119p*::*gon-1(sig_GON)]* animals. (i) Quantification of the fluorescence in neurons. The graph represents the combined results of 4 independent experiments (n > 20 neurons per strain). *p < 0.05. **p < 0.01. Bars represent the mean ± SE. Representative fluorescent images of INS-7::mCherry (j, k) and DIC images (inset) of the coelomocytes of wild-type (j) and *gon-1(tm3146)* mutant (k) animals (100 msec. exposure time). Coelomocytes are outlined with dotted circles in the fluorescent images. (l) Quantification of the fluorescence in coelomocytes. The graph represents the combined results of 4 independent experiments (n > 30 coelomocytes per strain). Fluorescence intensity was examined using ImageJ (NIH, Bethesda, MD). *p < 0.05. Bars represent the mean ± SE.

**Fig 3:**
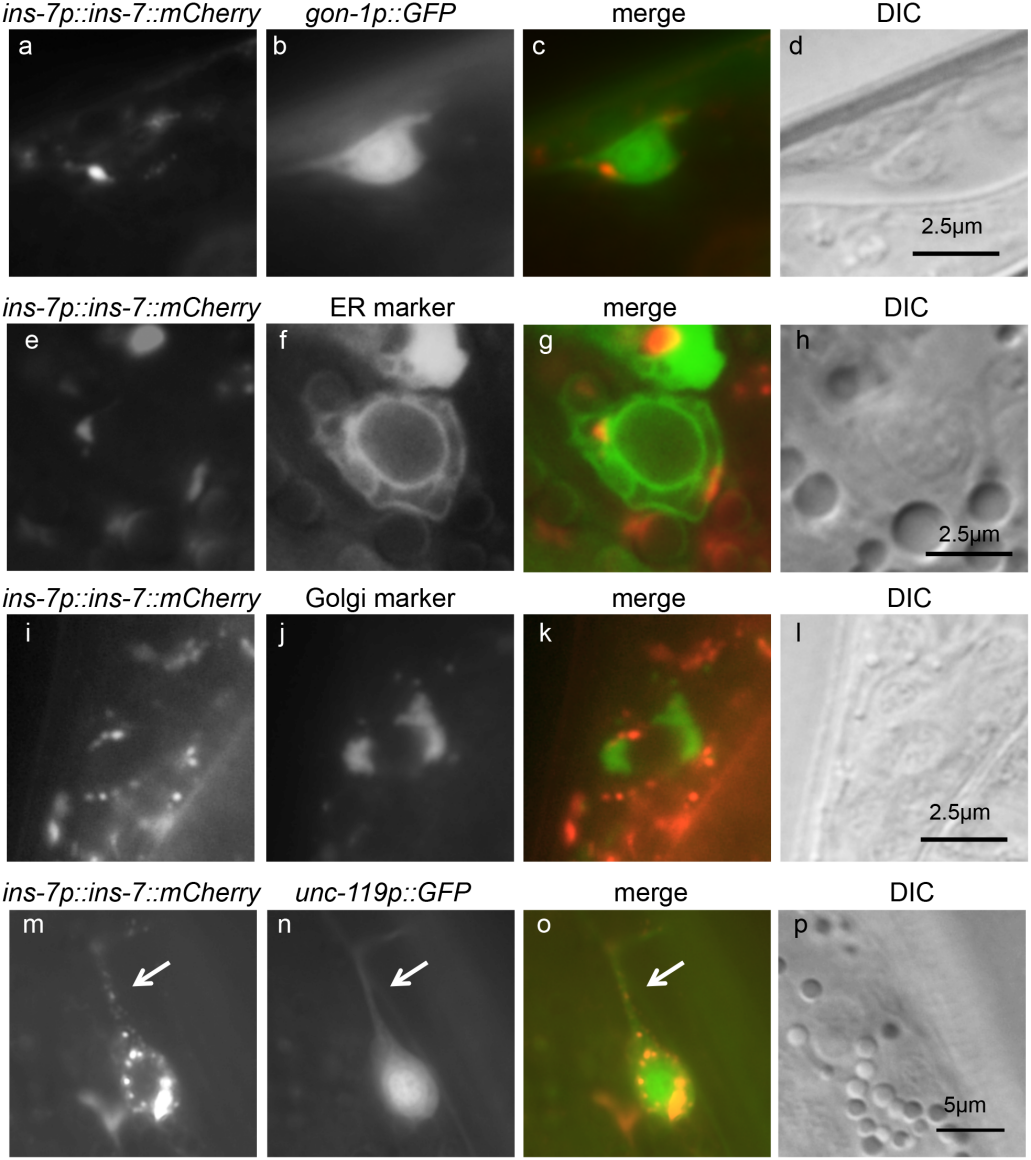
INS-7 accumulates in the ER and secretory vesicles in neurons in *gon-1* mutants. (a, b) Representative fluorescent images of a *gon-1(tm3146)* mutant animal expressing INS-7::mCherry (a) and *gon-1p*::*GFP* (b). (c) The merged image of (a) and (b). (d) The DIC image of the same field shown in (a-c). (e, f) Representative fluorescent images of mCherry-tagged INS-7 (e) and an ER marker (f) in a *gon-1* mutant animal. (g) The merged image. (h) The DIC image of the same field shown in (e-g). (i, j) Distribution patterns of mCherry-tagged INS-7 (i) and a Golgi marker (j) in a *gon-1* mutant animal. (k) The merged image. (l) The DIC image of the same field shown in (i-k). Scale bars indicate 2.5 μm. (m, n) Distribution patterns of mCherry-tagged INS-7 (m) and *unc-119p*::*GFP* (n) in a *gon-1* mutant animal. (o) The merged image. (p) The DIC image of the same field shown in (m-o). The arrow indicates an axon in which INS-7::mCherry is accumulated. The scale bar indicates 5 μm.

It has been reported that insulin secretion is reduced by a variant of ADAMTS9 that confers increased type 2 diabetes risks in humans. To assess whether GON-1 is involved in insulin secretion in *C*. *elegans*, we examined the effect of *gon-1* depletion on INS-7 secretion. We generated transgenic worms expressing an *ins-7* promoter-driven mCherry fused with INS-7. The accumulation of mCherry fluorescence was observed in some neurons in the *gon-1(tm3146)* mutant background (Fig 2c and 2i). Defects in protein secretion from the body wall muscle and DTC of *gon-1* mutants were rescued by the expression of the GON domain (*gon-1(sig_GON)*), which contains a signal sequence and the GON domain [12]. To investigate whether *gon-1(sig_GON)* could also rescue the accumulation of INS-7::mCherry, we generated transgenic animals expressing *gon-1* or pan-neuronal *unc-119* promoter-driven *gon-1(sig_GON)* in *gon-1(tm3146); tmIs1040[ins-7p*::*ins-7*::*mCherry]* worms and observed accumulation of INS-7::mCherry in neurons [14]. The accumulation of INS-7::mCherry was scarcely observed using transgenic animals that expressed *gon-1p*::*gon-1(sig_GON) or unc-119p*::*gon-1(sig_GON)* (Fig 2e, 2g and 2i). Under growth-promoting conditions, INS-7::mCherry accumulated in coelomocytes in wild-type animals at the adult stage, because coelomocytes specialize in taking up materials from the pseudocoelomic fluid [15]. By contrast, fluorescence intensity was decreased in coelomocytes of the *gon-1(tm3146)* mutant background (Fig 2j-2l). To examine whether *gon-1* and *ins-7* are expressed in the same cells, we generated transgenic animals co-expressing *ins-7p*::*ins-7*::*mCherry* and *gon-1p*::*GFP* in *gon-1(tm3146)* mutants. The accumulation of INS-7::mCherry was observed in some GFP-positive neuronal cells (Fig 3a-3d). To evaluate the localization of accumulated INS-7::mCherry in neurons, we generated transgenic animals co-expressing INS-7::mCherry and an ER marker (*unc-119* promoter-driven *cytb-5*.*1*::*GFP*), or a Golgi marker (*unc-119* promoter-driven *aman-2*::*GFP*) in *gon-1(tm3146)* mutants. INS-7::mCherry fluorescence was partially localized in the ER, as shown by the colocalization with the ER marker (Fig 3e-3h), but not in the Golgi (Fig 3i-3l). INS-7::mCherry was also localized in the axon (Fig 3m-3p, arrows).

**Fig 4:**
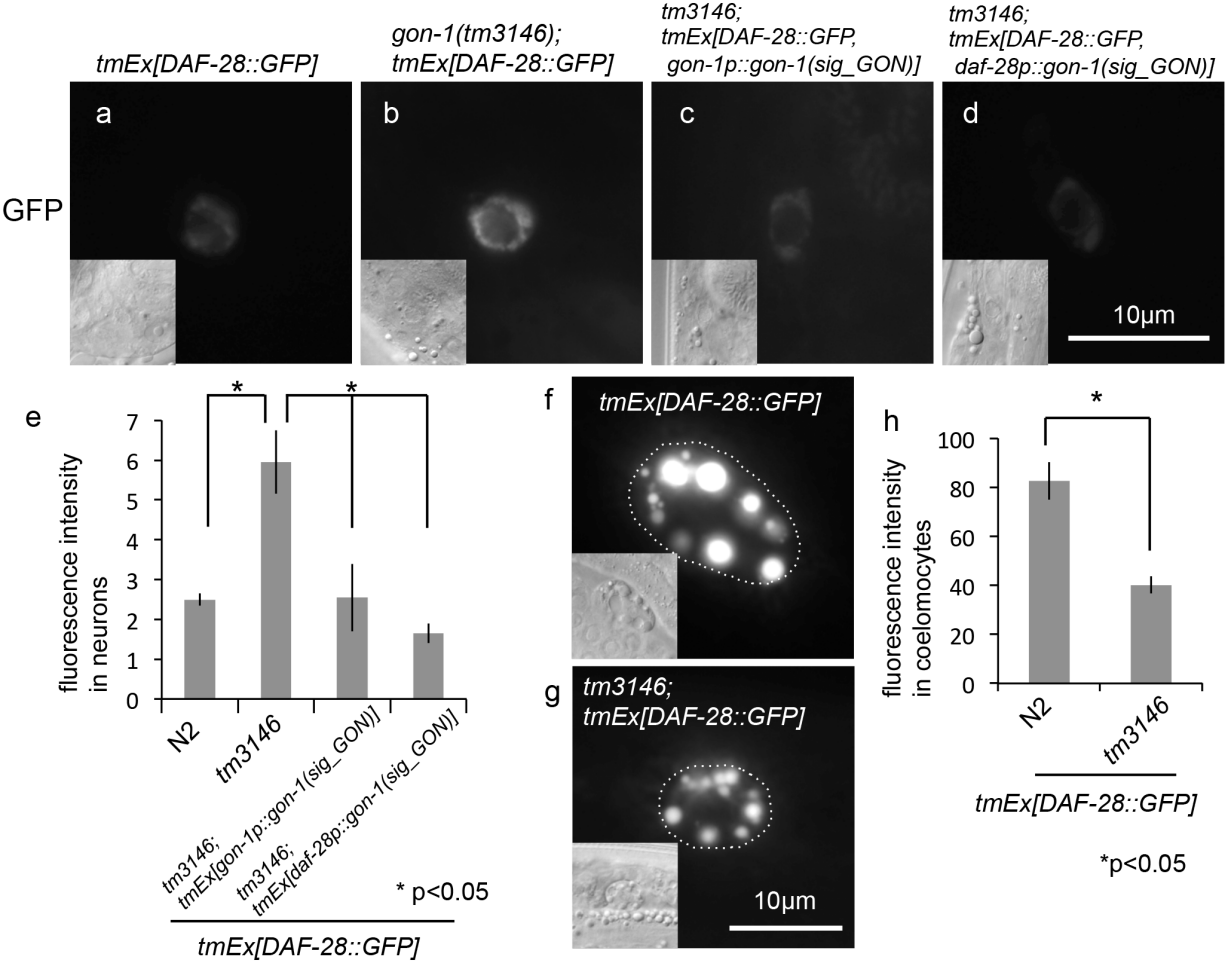
Depletion of *gon-1* causes DAF-28 secretion defects. (a-d) Fluorescent images of DAF-28::GFP, and DIC images (inset) in the head neurons of a wild-type animal (a), a *gon-1(tm3146)* mutant animal (b), *gon-1(tm3146)* mutant that expresses *gon-1p*::*gon-1(sig_GON)* (c) and *gon-1(tm3146)* mutant that expresses *daf-28p*::*gon-1(sig_GON)* (d) (500 msec. exposure time). The scale bar indicates 10 μm. (e) Quantification of fluorescence in neurons. The graph represents the combined results of 5 independent experiments (n > 20 neurons per strain). *p < 0.05. Bars represent the mean ± SE. (f, g) Representative fluorescent images of DAF-28::GFP in the coelomocytes of a wild-type animal (f) and a *gon-1(tm3146)* mutant animal (g) (100 msec. exposure time). Coelomocytes are outlined with dotted circles in the fluorescent images. The scale bar indicates 10 μm. (h) Quantification of fluorescence in coelomocytes. The graph represents the combined results of 5 independent experiments (n > 20 coelomocytes per strain). Fluorescence intensity was examined using ImageJ (NIH, Bethesda, MD). *p < 0.05. Bars represent the mean ± SE.

**Fig 5:**
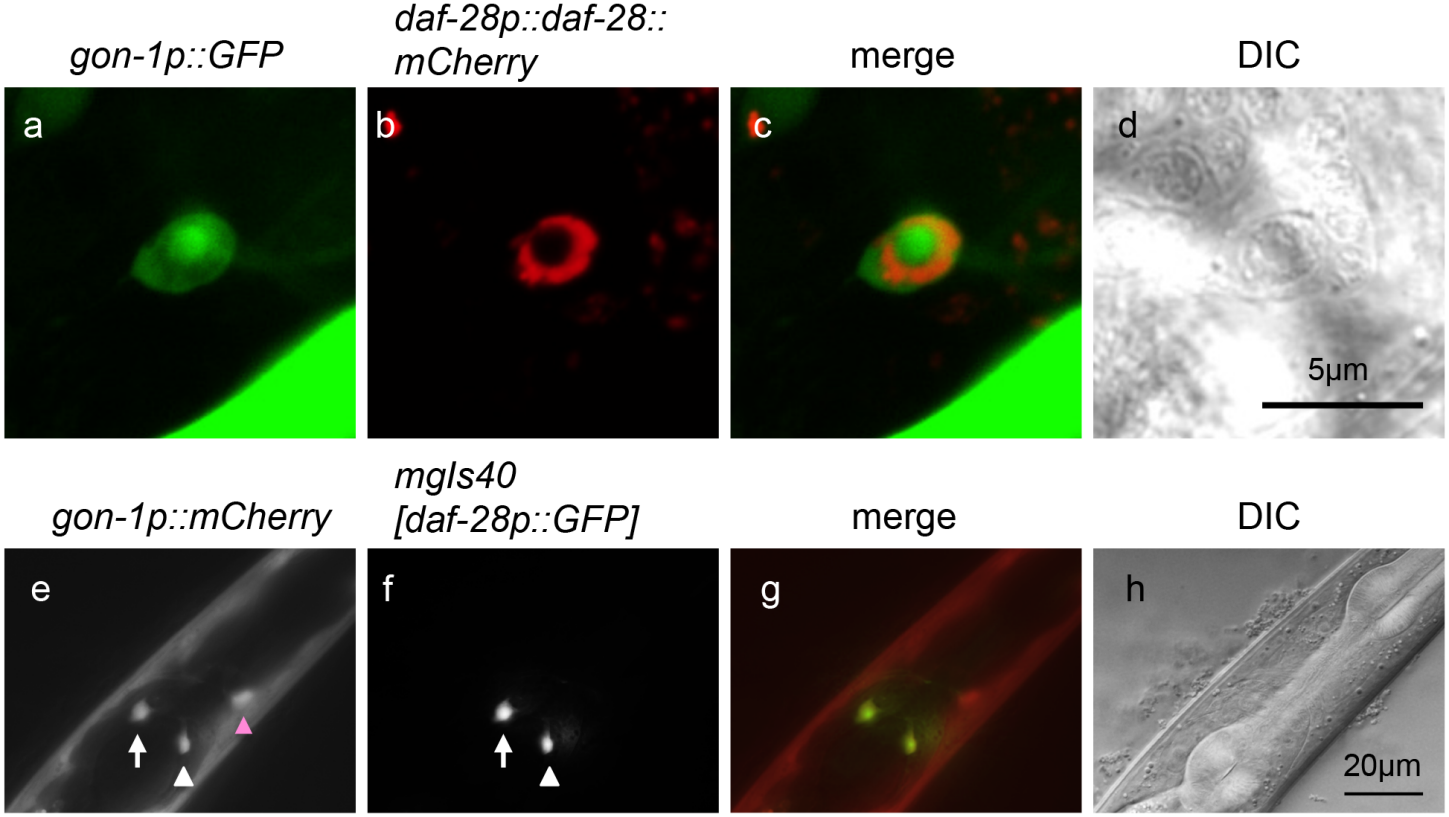
GON-1 and DAF-28 are expressed in the same cells. (a, b) Representative fluorescent images of a *gon-1(tm3146)* mutant animal co-expressing *gon-1p*::*GFP* (a) and *daf-28p*::*daf-28*::*mCherry* (b). (c) The merged image. (d) The DIC image of the same field shown in (a-c). The scale bar indicates 5 μm. (e, f) Representative fluorescent images of *gon-1p*::*mCherry* (e) and *mgIs40[daf-28p*::*GFP]* (f). (g) The merged image. (h) The DIC image of the same field shown in the (e-g). The scale bar indicates 20 μm. The white arrowhead indicates an ASI neuron. The white arrow indicates an ASJ neuron. The pink arrowhead indicates an unidentified neuron.

Because the *C*. *elegans* genome encodes 40 insulin-like peptides, we investigated the effect of *gon-1* depletion on the secretion of DAF-28, another insulin-like peptide. Mutations in *daf-28* cause dauer arrest [16]. DAF-28::GFP is expressed in ASI and ASJ neurons, secreted into the pseudocoelom, and taken up by the coelomocytes [16, 17]. As previously reported, under growth-promoting conditions, DAF-28::GFP was hardly detected in ASI and ASJ neurons (Fig 4a and 4e), and it accumulated in coelomocytes of wild-type animals at the adult stage (Fig 4f and 4h) [16, 17]. By contrast, the accumulation of GFP fluorescence was observed in some neurons in the *gon-1(tm3146)* mutant background (Fig 4b and 4e), whereas DAF-28::GFP fluorescence intensity was decreased in coelomocytes in the *gon-1(tm3146)* mutant background (Fig 4g and 4h). To investigate whether *gon-1(sig_GON)* could also rescue the accumulation of DAF-28::GFP, we generated transgenic animals co-expressing *gon-1* promoter-driven *gon-1(sig_GON)* and DAF-28::GFP or *daf-28* promoter-driven *gon-1(sig_GON)* and DAF-28::GFP in *gon-1(tm3146)* mutants and observed the accumulation of DAF-28::GFP in neurons. The accumulation of DAF-28::GFP was scarcely observed using transgenic animals that expressed *gon-1p*::*gon-1(sig_GON) or daf-28p*::*gon-1(sig_GON)* (Fig 4c, 4d and 4e). To examine whether *gon-1* and *daf-28* are expressed in the same cells, we generated transgenic animals co-expressing *daf-28p*::*daf-28*::*mCherry* and *gon-1p*::*GFP* in *gon-1(tm3146)* mutants (Fig 5a-5d). The accumulation of DAF-28::mCherry was observed in some GFP-positive neuronal cells. Next, we investigated whether *gon-1* is expressed in ASI and ASJ neurons. We generated transgenic animals expressing *gon-1p*::*mCherry* in *mgIs40[daf-28p*::*GFP]* worms [16]. *gon-1p*::*mCherry* and *daf-28p*::*GFP*, which are expressed in ASI and ASJ neurons, were observed in the same cells (Fig 5e-5h).

**Fig 6:**
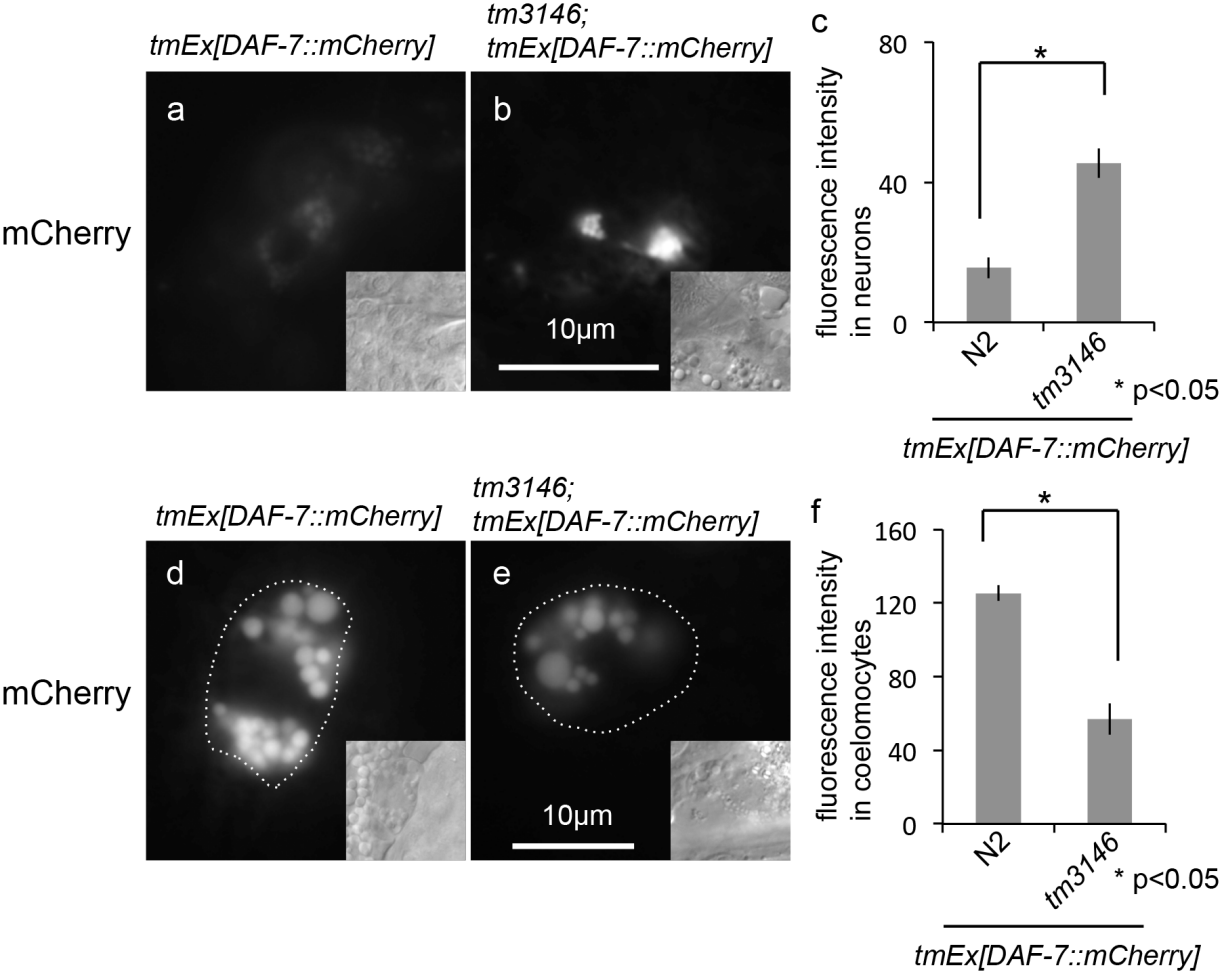
Depletion of *gon-1* causes DAF-7 secretion defects. (a, b) Fluorescent images of DAF-7::mCherry, and DIC images (inset) in the head neurons of a wild-type animal (a) and a *gon-1(tm3146)* mutant animal (b) (160 msec. exposure time). The scale bar indicates 10 μm. (c) Quantification of the mCherry fluorescence in neurons. The graph represents the combined results of 3 independent experiments (n > 20 neurons per strain). *p < 0.05. Bars represent the mean ± SE. (d, e) Representative fluorescent images of DAF-7::mCherry in the coelomocytes of wild-type (d) and a *gon-1(tm3146)* mutant (e) animals (100 msec. exposure time). Coelomocytes are outlined with dotted circles in the fluorescent images. Insets show DIC images. The scale bar indicates 10 μm. (f) Quantification of the mCherry fluorescence in coelomocytes. The graph represents the combined results of 3 independent experiments (n > 30 coelomocytes per strain). Fluorescence intensity was examined using ImageJ (NIH, Bethesda, MD). *p < 0.05. Bars represent the mean ± SE.

To determine whether GON-1 functions in more than insulin secretion, we assessed the effects of the *gon-1* mutation on DAF-7, another secretory protein. We investigated whether DAF-7 is normally secreted in the *gon-1* mutant background. *daf*-*7* encodes a TGF-β homolog that is secreted from ASI neurons. DAF-7::mCherry was hardly detected in ASI neurons (Fig 6a and 6c), and it accumulated in the coelomocytes of wild-type animals at the adult stage (Fig 6d and 6f). By contrast, the accumulation of mCherry fluorescence was observed in ASI neurons in the *gon-1(tm3146)* mutant background (Fig 6b and 6c) and DAF-7::mCherry fluorescence intensity was decreased in the coelomocytes of *gon-1(tm3146)* mutants (Fig 6e and 6f). Thus, GON-1 is necessary for the secretion of at least two major insulin peptides and TGF-β in *C*. *elegans*.

**Fig 7:**
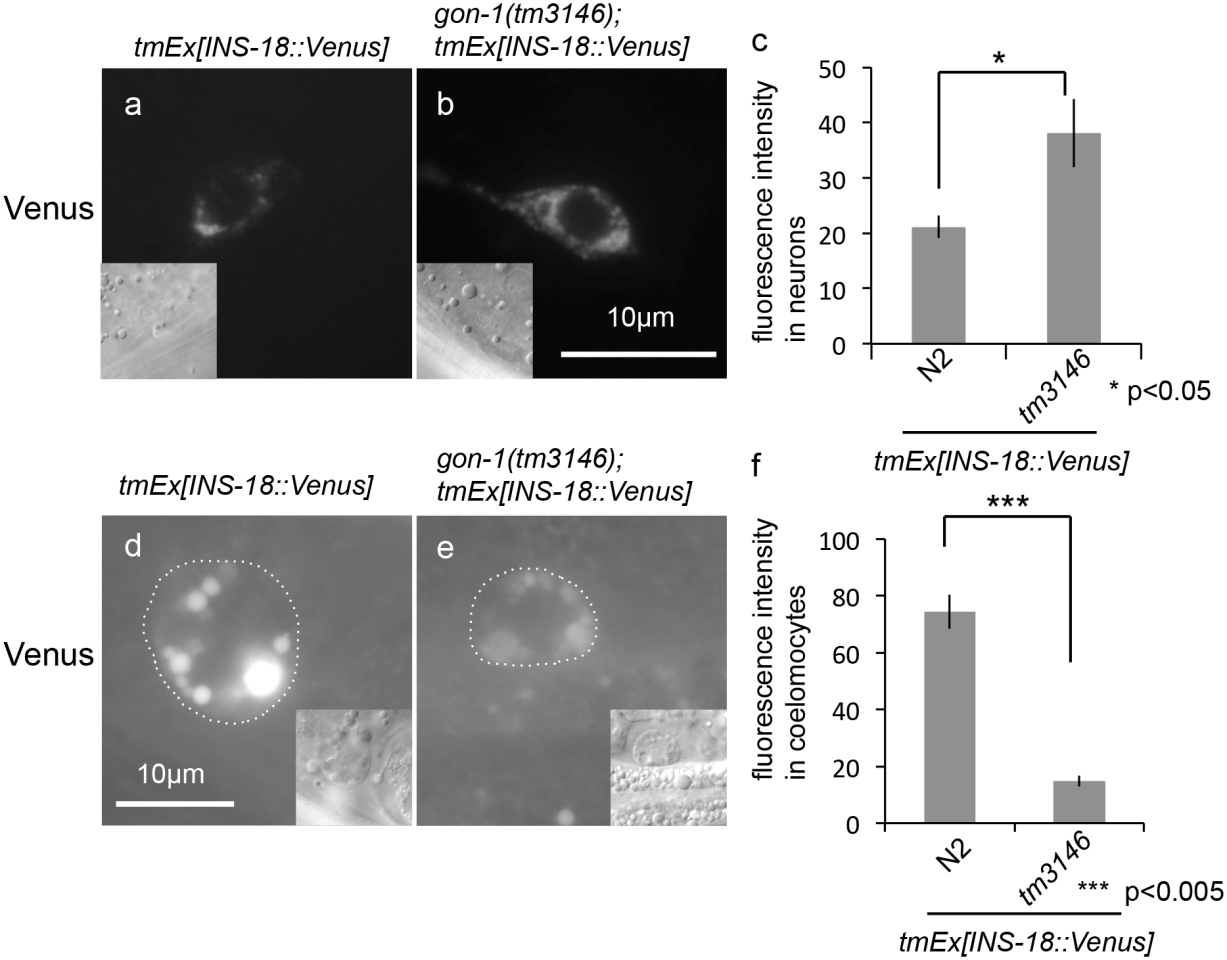
Depletion of *gon-1* causes INS-18 secretion defects. (a, b) Fluorescent images of INS-18::Venus, and (inset) DIC images in the head neurons of a wild-type animal (a), a *gon-1(tm3146)* mutant animal (b) (300 msec. exposure time). The scale bar indicates 10 μm. (c) Quantification of fluorescence in neurons. The graph represents the combined results of 3 independent experiments (n > 20 neurons per strain). *p < 0.05. Bars represent the mean ± SE. Representative fluorescent images of INS-18::Venus in the coelomocytes of a wild-type animal (d) and a *gon-1(tm3146)* mutant animal (e) (3 sec. exposure time). Coelomocytes are outlined with dotted circles in the fluorescent images. The scale bar indicates 10 μm. (f) Quantification of fluorescence in coelomocytes. The graph represents the combined results of 3 independent experiments (n > 20 coelomocytes per strain). Fluorescence intensity was examined using ImageJ (NIH, Bethesda, MD). ***p < 0.005. Bars represent the mean ± SE.

In *C*. *elegans*, insulins act as either DAF-2 agonists (such as INS-7 and DAF-28) or antagonists (INS-1 and INS-18) [18, 19]. We investigated the effect of *gon-1* depletion on the secretion of INS-18. Under growth-promoting conditions, INS-18::Venus was weakly detected in head neurons (Fig 7a and 7c), and it accumulated in the coelomocytes of wild-type animals at the adult stage (Fig 7d and 7f). By contrast, the accumulation of Venus fluorescence was observed in head neurons in the *gon-1(tm3146)* mutant background (Fig 7b and 7c), whereas INS-18::Venus fluorescence intensity was decreased in the coelomocytes of *gon-1(tm3146)* mutants (Fig 7e and 7f). Thus, secretory defects may not be specifically regulated between agonists and antagonists.

### GON-1 is required in the cells where insulin acts

**Fig 8:**
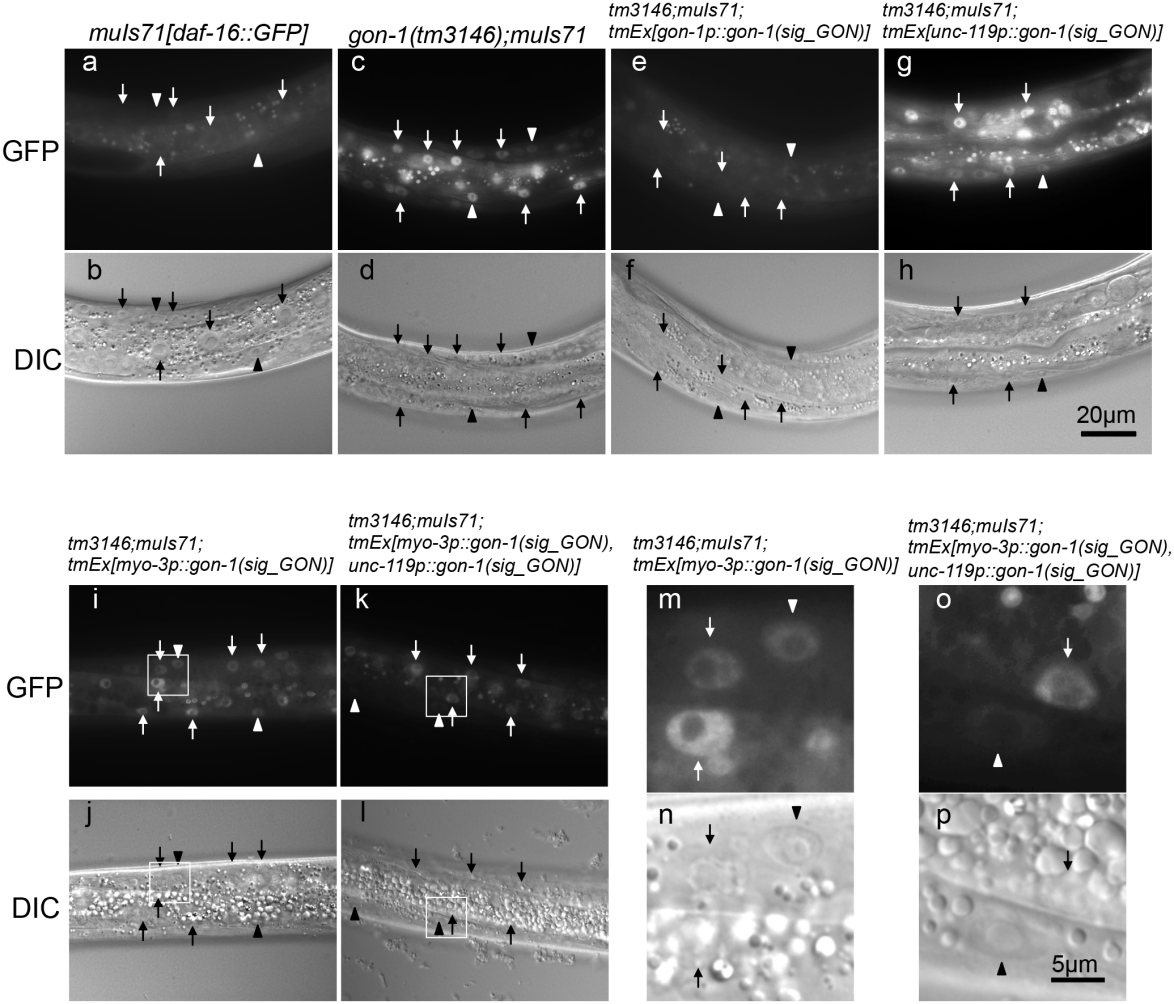
GON-1 acts for insulin signaling at peripheral tissues. Representative images of DAF-16a::GFP localization (a, c, e, g, i, k, m, o) and DIC (b, d, f, h, j, l, n, p) in wild-type (a, b), *gon-1(tm3146)* (c, d), *gon-1(tm3146); tmEx[gon-1p*::*gon-1(sig_GON)]* (e, f), *gon-1(tm3146); tmEx[unc-119p*::*gon-1(sig_GON)]* (g, h), *gon-1(tm3146); tmEx[myo-3p*::*gon-1(sig_GON)]* (i, j) and *gon-1(tm3146); tmEx[myo-3p*::*gon-1(sig_GON)*, *unc-119p*::*gon-1(sig_GON)]* (k, l) animals at the L2 stage. Arrowheads indicate nuclei of body wall muscle cells. Arrows indicate nuclei of other cells including intestinal cells. The scale bar indicates 20 μm for a-l. The white boxes marked in (i—l) are enlarged in (m—p). The scale bar indicates 5 μm.

To investigate the role of GON-1 in peripheral tissues, we next examined the subcellular localization of DAF-16/FOXO because the localization of FOXO in the nucleus is controlled by insulin signaling. DAF-16 has been shown to translocate from the nucleus to the cytoplasm by activating the insulin/IGF-like signaling pathway [20]. We used a strain harboring the transgene *muIs71*, an integrated DAF-16a::GFP construct [21], to study DAF-16 localization. As previously reported, DAF-16a::GFP was present in both the nucleus and the cytoplasm of wild-type animals (Fig 8a). By contrast, DAF-16a::GFP was exclusively localized to the nucleus in *gon-1(tm3146)* mutants (Fig 8c), and this phenotype was rescued by the expression of the GON domain under the control of the *gon-1* promoter as described above (Fig 8e). However, DAF-16a::GFP was localized to the nucleus in transgenic animals expressing either *unc-119p*::*gon-1(sig_GON)* (Fig 8g) or *myo-3p*::*gon-1(sig_GON)* (Fig 8i and 8m) in the *gon-1(tm3146)* mutant background. We inferred that DAF-16a::GFP localization requires both insulin secretion from neurons and signaling at peripheral cells. We generated transgenic animals expressing *myo-3-* and *unc-119-*promoter-driven *gon-1(sig_GON)* in *gon-1(tm3146); muIs71* worms and observed the localization of DAF-16a::GFP. DAF-16a::GFP was rarely observed in the nucleus of muscle cells (Fig 8k and 8o, arrowheads). However, DAF-16a::GFP was localized to the nucleus of cells excluding muscle cells (Fig 8k and 8o, arrows).

### GON-1 depletion alters dauer formation and lifespan

**Fig 9:**
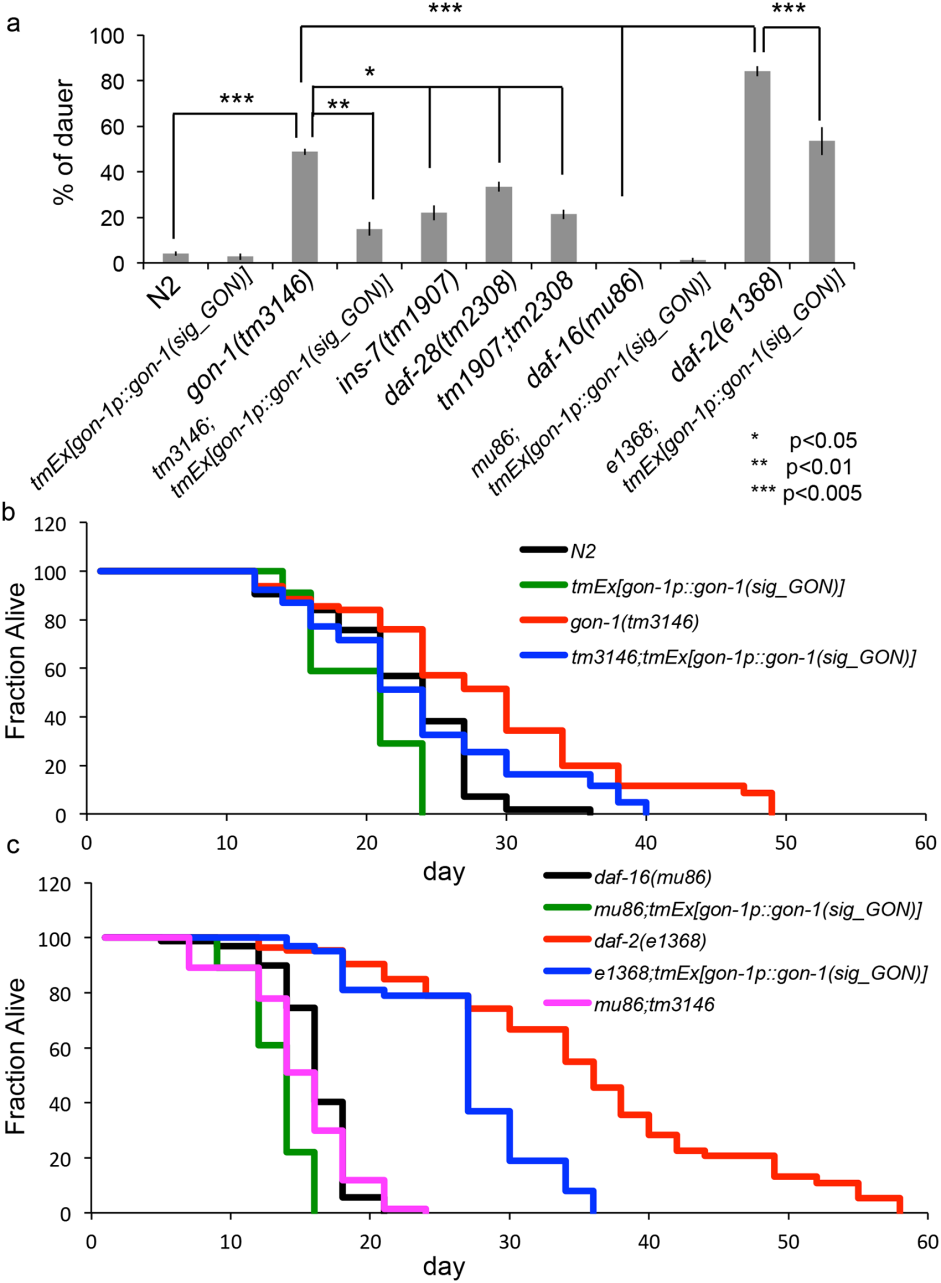
The GON domain expression alters dauer formation and longevity. (a) The percentage of dauer progeny from the indicated genotypes. *p < 0.05. **p < 0.01. ***p < 0.005. The graph represents the combined results of 3 independent experiments (n > 100 animals per strain). Bars represent the mean ± SE. (b) The percentage of animals remaining alive is plotted against animal age. Black, wild-type (N2) animals; green, *tmEx[gon-1p*::*gon-1(sig_GON)]* animals; red, *gon-1(tm3146)* mutants; blue, *gon-1(tm3146)* mutants expressing *gon-1p*::*gon-1(sig_GON)*. N2: n = 64. *tmEx[gon-1p*::*gon-1(sig_GON)]*: n = 50. *gon-1(tm3146)*: n = 74. *gon-1(tm3146);tmEx[gon-1p*::*gon-1(sig_GON)]*: n = 51. Nineteen *gon-1(tm3146)* mutants died with a burst vulva phenotype from day 0 to day 16 and were excluded. (c) The percentage of animals remaining alive is plotted against animal age. Black, *daf-16(mu86)* animals*;* green, *daf-16(mu86); tmEx[gon-1p*::*gon-1(sig_GON)]* animals*;* red, *daf-2(e1368)* animals*;* blue, *daf-2(e1368); tmEx[gon-1p*::*gon-1(sig_GON)]* animals; magenta, *daf-16(mu86);gon-1(tm3146)*. *daf-16(mu81)*: n = 78. *daf-16(mu86);tmEx[gon-1p*::*gon-1(sig_GON)]*: n = 90. *daf-2(e1368)*: n = 56. *daf-2(e1368); tmEx[gon-1p*::*gon-1(sig_GON)]*: n = 56. *daf-16(mu86);gon-1(tm3146)*: n = 69.

In *C*. *elegans*, it is well established that the insulin/IGF-like signaling pathway controls dauer arrest by regulating the subcellular localization of DAF-16. The reduction of insulin/IGF-like signaling results in constitutive dauer arrest [22]. We examined the effect of *gon-1* depletion on the dauer-arrest phenotype. Under the starvation condition, about half of *gon-1* mutants became dauers, and this phenotype was partially rescued by GON domain expression under the control of the *gon-1* promoter (Fig 9a). Thus, the GON domain is important for dauer formation. We compared the effect of *gon-1(tm3146)*, *ins-7(tm1907)*, *daf-28(tm2308)*, and *daf-2(e1368)* single mutations and the *ins-7(tm1907); daf-28(tm2308)* double mutation on the dauer-arrest phenotype. *gon-1(tm3146)* mutants had a stronger dauer-constitutive (Daf-c) phenotype than *ins-7(tm1907)* or *daf-28(tm2308)* single mutants or *ins-7(tm1907);daf-28(tm2308)* double mutants. *daf-2(e1368)* mutants had a stronger Daf-c phenotype than *gon-1(tm3146)*. To investigate whether overexpression of GON domain alters dauer arrest in Daf-c and dauer-defective (Daf-d) mutants, we generated transgenic animals that express the GON domain under the control of the *gon-1* promoter in wild type, *daf-16(mu86)* and *daf-2(e1368)* mutants. GON domain expression reduced dauer arrest in the *daf-2(e1368)* mutant background. By contrast, there were no significant differences between N2 and *tmEx[gon-1p*::*gon-1(sig_GON)]* worms or *daf-16(mu86)* and *daf-16(mu86);tmEx[gon-1p*::*gon-1(sig_GON)]* worms (Fig 9a).

Because *C*. *elegans* adult lifespan is extended by reduced insulin signaling, we next investigated whether *gon-1* affects longevity. *gon-1(tm3146)* mutants exhibited an extended lifespan relative to wild-type animals, whereas the lifespan of *gon-1(tm3146);tmEx[gon-1p*::*gon-1(sig_GON)]* animals was similar to or slightly longer than that of wild-type animals, shorten than that of *gon-1(tm3146)* mutant animals. Expression of the GON domain shortened the lifespan of wild-type animals (Fig 9b and S2 Table). We next investigated whether overexpression of the GON domain alters lifespan in Daf-c and Daf-d mutants. The lifespan of *daf-16 (mu86)* and *daf-2(e1368)* mutants was shortened by expression of the GON domain under the control of the *gon-1* promoter (Fig 9c and S2 Table). Moreover, we investigated the effect of *gon-1* depletion in *daf-16(mu86)* mutants and found that *gon-1(tm3146)* has no effect on the lifespan of *daf-16* mutants (Fig 9c and S2 Table).

## Discussion

### The GON domain is required at insulin secretory cells and peripheral tissues for normal insulin signaling

GON-1 belongs to the ADAMTS protease family, which functions in ECM remodeling in *C*. *elegans*. Depletion of GON-1 results in gonadal proliferation defects and aberrant membrane morphology in hermaphrodite DTCs [13]. We found that GON-1 is required for the protein secretory pathway from the ER to the Golgi [12]. In the present study, we show that the insulin/IGF-like signaling pathway is compromised by the depletion of *gon-1* in *C*. *elegans*. The insulin secretion defect in *gon-1* mutants was restored by GON domain expression under the control of either the *gon-1* promoter or the pan-neuronal *unc-119* promoter. However, localization of DAF-16a::GFP to peripheral tissues was not restored by expression of either *unc-119p*::*gon-1(sig_GON)* nor *myo-3p*::*gon-1(sig_GON)* in the *gon-1(tm3146)* mutant background. Proper localization of DAF-16a::GFP in the whole body was restored by GON domain expression under the *gon-1* promoter. The localization of DAF-16a::GFP was restored only in the body wall muscles by co-expressing *unc-119p*::*gon-1(sig_GON)* and *myo-3p*::*gon-1(sig_GON)* in the *gon-1(tm3146)* mutant background. This result indicates that the expression of *myo-3p*::*gon-1(sig_GON)* allowed peripheral tissues to receive insulin, but the insulin secretion defect was not restored in *gon-1(tm3146); tmEx[myo-3p*::*gon-1(sig_GON)]* transgenic animals. Therefore, DAF-16a::GFP was localized to the nucleus in these animals. Insulin is normally secreted from neurons, and received at the body wall muscle cells in *gon-1(tm3146); tmEx[unc-119p*::*gon-1(sig_GON)*, *myo-3p*::*gon-1(sig_GON)]* transgenic animals. Consequently, DAF-16a::GFP was not localized to the nucleus at body wall muscle cells, leaving DFA-16a::GFP localized to nuclei in other cells. These data suggest that the GON domain alone, but not the protease domain, is required for insulin secretion and signaling. In addition, our data suggest that expression of GON-1 at insulin secretory cells and peripheral tissues is important for normal insulin signaling.

It has been reported that the DAF-2/DAF-16-mediated insulin signaling pathway is coordinated among different tissues by positive-feedback regulation of an insulin-like peptide [14, 23]. We speculate that this positive feedback loop in insulin signaling by INS-7 is blocked in the *gon-1(tm3146)* mutant background. We have reported that knockdown of ADAMTS9/GON-1 inhibited membrane protein transport [12]. The data presented in this paper raises the possibility that GON-1 depletion in peripheral tissues might impair the transport of membrane proteins that are required for the insulin/IGF-like signaling pathway, thereby resulting in insulin resistance. Future studies are necessary to confirm that the insulin receptor and glucose transporters, which are related to insulin resistance, are indeed functionally inhibited by GON-1 depletion. In mammals, insulin regulates glucose uptake in peripheral tissues by promoting the translocation of GLUT4 to the plasma membrane [24]. It has been reported that glucose levels change in response to insulin/IGF-like signaling in *C*. *elegans* [25]. However the *C*. *elegans* genome encodes members of the glucose transporter superfamily, there is no evidence which glucose transporter is regulated by insulin [26]. It would be interesting to determine which molecules account for this phenomenon.

### Overexpression of GON domain alters dauer formation and longevity

In our current experiments, we showed that depletion of GON-1 induced dauer formation and longevity. Surprisingly, the dauer formation phenotype was stronger in *gon-1* mutant animals than in *ins-7* or *daf-28* mutants. Consistent with this observation, DAF-7, another protein involved in dauer formation, was also inefficiently secreted (Fig 6). Thus, dauer formation was more strongly induced by the depletion of GON-1 than by the depletion of INS-7 and DAF-28. In addition, *gon-1* mutants suffer from Unc phenotypes (data not shown) by secretion defects. Unc phenotypes cause a nutrition uptake defect, and may augment dauer phenotype. Thus, *gon-1* mutants may tend to form dauers and show extended longevity.

The Daf-c phenotype of *gon-1(tm3146)* mutants was partially rescued by GON domain expression under the control of the *gon-1* promoter. The lifespan of *gon-1* mutants was shortened by GON domain expression. Moreover, the Daf-c phenotype of *daf-2(e1368)* was suppressed by expression of the GON domain. The lifespans of *daf-2(e1368)* and *daf-16(mu86)* worms were shortened by expression of the GON domain. These data suggest that the amount of secreted insulin may be increased by overexpression of the GON domain, and consequently dauer formation and lifespan may be affected. It was reported that overexpression of INS-7 in the intestine shortens lifespan [14]. Surprisingly, expression of the GON domain shortened the lifespan of *daf-16(mu86)* mutants. This result indicates that the GON domain may also alters DAF-16- independent molecules such as extracellular matrix proteins, to control lifespan [27].

### A model of insulin resistance and insulin secretion defects

It has been reported that the gene encoding *O*-linked-*N*-acetyl glucosaminidase (OGA), MGEA5, is linked to insulin resistance, a hallmark of type 2 diabetes [28–30]. In *C*. *elegans*, knockout mutants of the *oga-1*, a homolog of human OGA, decrease fat stores and exhibit resistance to the insulin-like signaling pathway [31, 32]. The knockout mutant of *oga-1* was proposed as a genetic model of insulin resistance in peripheral tissues. Our data suggest that GON-1 is involved directly or indirectly both in insulin secretion by affecting insulin secretory cells and insulin signaling by affecting peripheral tissues. The *gon-1* mutant may be a model of both insulin secretion from insulin secretory cells and insulin resistance in peripheral tissues. It has been reported that a variant of ADAMTS9 is associated with type 2 diabetes [1]. One study reported that the diabetes risk allele of ADAMTS9 was associated with reduced insulin sensitivity but not with insulin secretion [33]. Another study reported that the diabetes risk allele of ADAMTS9 was associated with impaired beta cell function [34]. However, the molecular mechanisms through which ADAMTS9 affects insulin sensitivity and/or secretion in the diabetes risk variant are unknown. Although future studies are needed to conclude whether ADAMTS9 is involved both in insulin secretion by beta cells and insulin signaling at peripheral tissues in humans, considering the similar functions of the GON domains in worms and humans, it is highly possible that ADAMTS9 is involved in both mechanisms.

In summary, we show that the GON domain of GON-1 is involved in protein secretion from insulin secretory cells and in insulin/IGF-like signaling at peripheral tissues. GON-1 may be involved in transport of membrane proteins that are required for the insulin/IGF-like signaling pathway at peripheral tissues. Moreover, at least in *C*. *elegans*, the GON domain may play an important role in the insulin feedback loop.

## Materials and Methods

### Nematode strains

*C*. *elegans* strain N2 worms were used as wild-type animals. Worms were grown at 20°C under well-fed conditions, using standard methods [35]. The deletion mutant strains *gon-1(tm3146)*, *ins-7(tm1907)* and *daf-28(tm2308)* were obtained as described [36]. The primers used in nested PCR screening for detecting *gon-1(tm3146)* were as follows: first round: 5'-GGGTGTTGTGTAATAGCCCA-3', 5'-TCCGTCGGCCCATGGCATAT-3'; second round: 5'-GTGGGTCCCCTATAGCTAAT-3', 5'- AGCCCATCTGGCTTCCGTAG-3'. The primers used in nested PCR screening for detecting *ins-7(tm1907)* were as follows: first round: 5'-CATAGCGCGAGATCAGTTTG-3', 5'-CCAGAGAATCAGCCGCTCGA-3'; second round: 5'-CGAGATCAGTTTGCGTAGCT-3', 5'-GTCTGCAATCGCGGCTAATG-3'. The primers used in nested PCR screening for detecting *daf-28(tm2308)* were as follows: first round: 5'-TCCGCCCACTTTGAGCTATA-3', 5'-GCACCCGATCTGACGACACT-3'; second round: 5'-GGGTTATCACTAGGAAGTTG-3', 5'-ACCGAGAGGTAGGGGTAATT-3'. *daf-16(mu86)*; *muIs71*[*pKL99*(*daf-16a*::*GFP/bKO*)+*pRF4(rol-6)*]X, *daf-2(e1368)*, *ctIs40[pTG96 (sur-5*::*gfp)]*, and *mgIs40[daf-28p*::*GFP]* were provided by the Caenorhabditis Genetics Center.

### Generation of transgenic strains

To construct *gon-1p*::*gon-1(sig_GON)*, the promoter region of *gon-1* was amplified by PCR from *C*. *elegans* genomic DNA using the primers 5'-GAAATGAAATAAGCTTGTCAGAATGAACAAAGGGGGT-3' and 5'-GCAGGCATGCAAGCTTACCAACAGCTCCGTGATGATG-3' and inserted into the pPD95.79 vector plasmid (a gift from Dr. A. Fire), yielding *pPD95*.*79_gon-1p*.

To construct *unc-119p*::*gon-1(sig_GON)*, the promoter region of *unc-119* was amplified by PCR from *C*. *elegans* genomic DNA using the primers 5'-GAAATGAAATAAGCTTGTGCCAAGCTTCAGTAAAAGAAG-3' and 5'-GCAGGCATGCAAGCTTTATATGCTGTTGTAGCTGAAAA-3' and inserted into the pPD95.79 vector plasmid, yielding *pPD95*.*79_unc-119p*. The cDNA of *gon-1(sig_GON)* was amplified by PCR using the primers 5'-GTAAGCTTGCATGCCTGCAGATGCGCTCCATCGGCGGCTCATT-3' and 5'-CCTCTAGAGTCGACCTGCAGTTAATCCATGTCACCAGAGAAAC-3', with *pPD95*.*79_lag-2p*::*gon-1(sig_GON)* as the template [12]. The fragment was inserted into the plasmids *pPD95*.*79_gon-1p* and *pPD95*.*79_unc-119p* using the *Pst*I site.

To construct *ins-7p*::*ins-7*::*mCherry*, the mCherry fragment was inserted into the *pFX_VT* vector plasmid using the *Not*I and *Bgl*II sites, giving rise to *pFX_VT_Venus* [37], yielding *pFX_VT_mCherry*. The promoter and genomic region of *ins-7* was amplified from *C*. *elegans* genomic DNA using the primers 5'-GGTTCCGCGTGGATCCTTTTGCTTCGAAGGATAACCCCG-3' and 5'- GCTCACCATGCGGCCGCAAGGACAGCACTGTTTTCGAATG-3'. The fragment was inserted into the plasmid *pFX_VT_mCherry* using the *Not*I and *BamH*I sites.

To construct *unc-119p*::*cytb-5*.*1*::*GFP* (an ER marker), the cDNA fragment of *cytb-5*.*1* (*C31E10*.*7)* was amplified by PCR using the primers 5'-CGACTCTAGAGGATCCATGGCCGATCTTAAGCAAATCA-3' and 5'-CCAATCCCGGGGATCCGCAGCGATAAGATAATAAACA-3', with *lag-2p*::*C31E10*.*7*::*Venus* as the template [12]. The *C31E10*.*7* cDNA fragment was inserted into the plasmid *pPD95*.*79_unc-119p* using the *BamH*I site.

To construct *unc-119p*::*aman-2*::*GFP* (a Golgi marker), the *aman-2* cDNA fragment (1-82aa) was amplified by PCR using the primers 5'-CGACTCTAGAGGATCCATGGGAAAACGCAATTTCTATA-3' and 5'-CCAATCCCGGGGATCCTCTTTTTCTTCATCAAAATCTAC-3', with *lag-2p*::*aman-2*::*Venus* as the template [12]. The *aman-2* cDNA fragment (1-82aa) was inserted into the plasmid *pPD95*.*79_unc-119p* using the *BamH*I site.

To construct *daf-28p*::*daf-28*::*GFP*, the promoter and genomic region of *daf-28* was amplified from *C*. *elegans* genomic DNA using the primers 5'-GAAATGAAATAAGCTTTGGTAGGATAGTTGGTTGCCGG-3' and 5'- CCAATCCCGGGGATCCAGAAGCAAACGTGGGCAACAGGCAG-3'. The fragment was inserted into the plasmid pPD95.75 using the *Hind*III and *BamH*I sites.

To construct *daf-28p*::*gon-1(sig_GON)*, the promoter of *daf-28* was amplified from *C*. *elegans* genomic DNA using the primers 5'-CTCACAGTGGTCTAGATGGTAGGATAGTTGGTTGCCGG-3' and 5'- GCATGGATCCTCTAGAGTTGAGATAGTTGTTGAGAGG-3'. The fragment was inserted into the plasmid *pPD95*.*79_gon-1(sig_GON)* using the *Xba*I site.

To construct *daf-28p*::*daf-28*::*mCherry*, the mCherry fragment was inserted into the *pPD95*.*79* vector plasmid using the *Kpn*I and *EcoR*I sites, yielding *pPD95*.*79_mCherry*. The promoter and genomic region of *daf-28* was amplified from *C*. *elegans* genomic DNA using the primers 5'-GGTTCCGCGTGGATCCTTTTGCTTCGAAGGATAACCCCG-3' and 5'-CCCTTGCTCACCATGGTACCAAGAAGCAAACGTGGGCAACAG-3'. The fragment was inserted into the plasmid *pPD95*.*79_mCherry* using the *Hind*III and *Kpn*I sites.

To construct *daf-7p*::*daf-7*::*mCherry*, the promoter and genomic region of *daf-7* was amplified from *C*. *elegans* genomic DNA using the primers 5'-GAAATGAAATAAGCTTAACCCTCGAATTCCCGTTCCATC-3' and 5'- CCCTTGCTCACCATGGTACCTGAGCAACCGCATTTCTTGGCG-3'. The fragment was inserted into the plasmid *pPD95*.*79_mCherry* using the *Hind*III and *Kpn*I sites.

To construct *gon-1p*::*mCherry*, the promoter of *gon-1* was amplified from *C*. *elegans* genomic DNA using the primers 5'-GAAATGAAATAAGCTTGTCAGAATGAACAAAGGGGGT-3' and 5'- CCCTTGCTCACCATGGTACCACCAACAGCTCCGTGATGATG-3'. The fragment was inserted into the plasmid *pPD95*.*79_mCherry* using the *Hind*III and *Kpn*I sites.

To construct *myo-3p*::*gon-1(sig_GON)*, the cDNA of *gon-1(sig_GON)* was amplified by PCR using the primers 5'-TGACTAGTGGCGGCCGCATGCGCTCCATCGGCGGCTCATTCCATC-3' and 5'-TCAAAAATAGAGATCTTTAATCCATGTCACCAGAGAAACCATCATC -3', with *pPD95*.*79_lag-2p*::*gon-1(sig_GON)* as the template [12]. The fragment was inserted into the plasmid *pFX_myo-3p*::*VenusT* [37] using the *Not*I and *Bgl*II sites.

To generate transgenic animals expressing an *ins-18* reporter gene, we used the same plasmid construct, *ins-18p*::*ins-18*::*Venus*, used by [19].

The micro-injection of the DNA constructs described above was performed as previously reported [38] with appropriate selection markers. The transgenic strains used in this work are listed in S1 Table. Images were taken with a BX-51 microscope (Olympus, Japan) or a LSM710 confocal laser-scanning microscope (Zeiss, Germany).

### Measurement of fluorescence intensity in coelomocytes and neurons

Images were taken with a BX-51 microscope (Olympus, Japan). Exposure times were described at each figure legend. Data of fluorescence intensity in coelomocytes and neurons were collected and scored by a blinded observer.

### Assays for the Daf phenotype

Dauer assays were performed similarly to those previously described [39]. Approximately 100–200 eggs of each genotype were placed on seeded 3.5 cm NGM agar plates and allowed to starve at 25°C. Four days after the food was exhausted, the plates were flooded with 1% SDS solution to select for surviving dauers. After 10–15 min, the plates were scored visually for dauers by using a microscope.

### Lifespan analysis

Lifespan assays were performed as described previously [40–42]. Briefly, synchronous animal populations were generated by hypochlorite treatment of gravid adults. Late L4 larvae growing at 20°C were transferred to NGM plates with FUdR (40 μM) and incubated at 20°C. In lifespan assays, the first day of adulthood was defined as day 1. Nematodes that failed to display touch-provoked movement were scored as dead. Nematodes that died from causes other than aging, such as sticking to the plate walls or gonadal extrusion were censored as lost worms. Statistical analyses of lifespan were performed on Kaplan-Meier survival curves in Prism 6 by Logrank (Mantel-Cox) tests (S2 Table).

## Supporting Information

S1 Fig Expression patterns of *gon-1p*::*GFP*. (a, c, e, g), GFP fluorescence; (b, d, f, h), DIC images. *gon-1* is expressed in motor neurons at the adult stage (a), intestine at the L4 stage (c), vulval muscle, VC4 and VC5 at the adult stage (e). The white arrow indicates VC4. The white arrowhead indicates VC5. The asterisk indicates vulval muscle. *gon-1* is also expressed in some cells at the embryonic stage (260–280 min after the first cleavage) (g). The scale bars indicate 20 μm.
(TIF)

S1 Table Transgenic strains used in this work. (XLSX)

S2 Table Detailed parameters of adult lifespan of each genotype. † The number of animals scored in Fig 9b and 9c. †† The number of animals that were censored because they had crawled off the plate or were exploded. *** p < 0.005; **** p < 0.0001; ns, not significant.
(XLSX)
