# Supplementary figures and images for: Loss of C. elegans GON-1, an ADAMTS9 Homolog, Decreases Secretion Resulting in Altered Lifespan and Dauer Formation
Source: PLoS One. 2015 Jul 28;10(7):e0133966. doi: 10.1371/journal.pone.0133966 (PMC4517882; doi:10.1371/journal.pone.0133966)

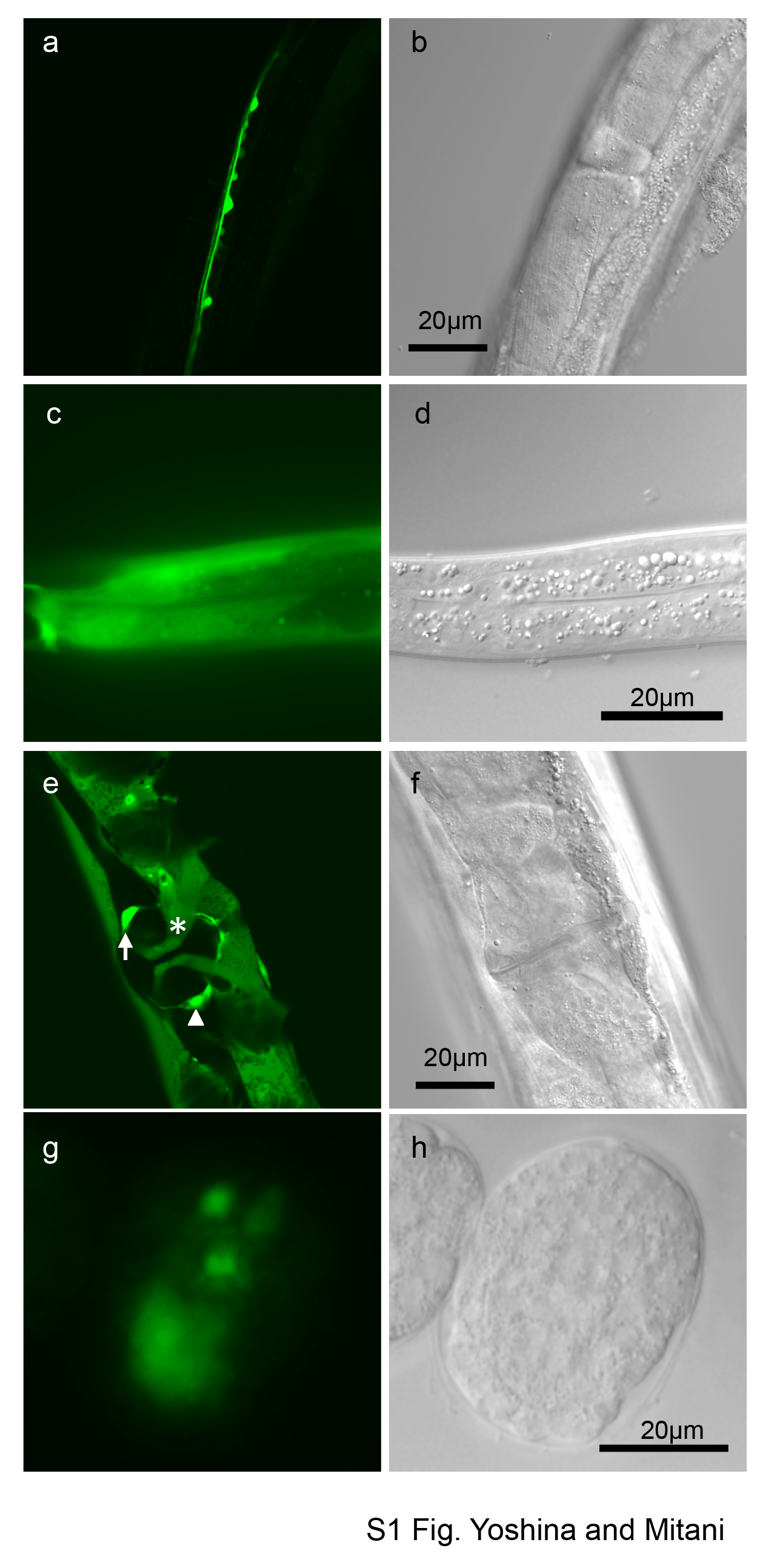

Supplement: S1 Fig — (a, c, e, g), GFP fluorescence; (b, d, f, h), DIC images. gon-1 is expressed in motor neurons at the adult stage (a), intestine at the L4 stage (c), vulval muscle, VC4 and VC5 at the adult stage (e). The white arrow indicates VC4. The white arrowhead indicates VC5. The asterisk indicates vulval muscle. gon-1 is also expressed in some cells at the embryonic stage (260–280 min after the first cleavage) (g). The scale bars indicate 20 μm. (TIF) [file pone.0133966.s001.tif]
